# Regulatory Actions of LH and Follicle-Stimulating Hormone on Breast Cancer Cells and Mammary Tumors in Rats

**DOI:** 10.3389/fendo.2018.00239

**Published:** 2018-05-16

**Authors:** Angel Matias Sanchez, Marina Ines Flamini, Sara Zullino, Eleonora Russo, Andrea Giannini, Paolo Mannella, Antonio Giuseppe Naccarato, Andrea Riccardo Genazzani, Tommaso Simoncini

**Affiliations:** ^1^Molecular and Cellular Gynecological Endocrinology Laboratory (MCGEL), Department of Clinical and Experimental Medicine, University of Pisa, Pisa, Italy; ^2^Laboratorio de Transducción de Señales y Movimiento Celular, Instituto de Medicina y Biología Experimental de Cuyo (IMBECU), Consejo Nacional de Investigaciones Científicas y Técnicas (CONICET), Mendoza, Argentina; ^3^Laboratorio de Biología Tumoral, Instituto de Medicina y Biología Experimental de Cuyo (IMBECU), Consejo Nacional de Investigaciones Científicas y Técnicas (CONICET), Mendoza, Argentina; ^4^Department of Translational Research and of New Surgical and Medical Technologies, University of Pisa, Pisa, Italy

**Keywords:** LH, follicle stimulating hormone, moesin and focal adhesion kinase, cell motility, breast cancer

## Abstract

Gonadotrophins are mainly known to influence the body through the formation of gonadal steroids. However, receptors for luteinizing hormone (LH) and follicular-stimulating hormone (FSH) are present in a set of extra-gonadal tissues in humans and animals, but their functional relevance is uncertain. In this article, we present experimental evidence that, in T-47D breast cancer (BC) cells, FSH, and LH alter the expression of genes involved in adhesion, motility, and invasion through the activation of their receptors. Using miniarray technology we also found that LH influences the expression of a broad set of genes involved in cancer biology in T-47D cells. Interestingly, the regulatory actions of FSH and LH depend on the modality of exposure, with significant differences between pre-pubertal-like vs. post-menopausal-like amounts of gonadotrophins, but not after intermittent administration, representative of fertile life. We also studied the modulation of the circulating levels of gonadotrophins in an *in vivo* rat model of BC progression and observed a direct correlation with the extent of cancer growth. These results support the hypothesis that gonadotrophins may have direct effects on extra-gonadal tissues. They also highlight that gonadotrophins could potentially contribute to BC progression, particularly in post-menopausal women who typically have higher gonadotrophin levels. This research may ultimately lead to testing the use of gonadotrophin-modulating drugs in BC patients.

## Introduction

In Western countries, breast cancer (BC) is the most prevalent cancer in women. In 2017, it was expected that over 252,710 women would be diagnosed with BC in the United States (1). BC is a leading cause of death due to the metastatic process (2). In spite of the technological advancements in diagnosis and treatment, new strategies as well as new molecular targets are urgently needed to fight against BC.

Hormone-dependent BCs account for 80% of diagnosed cases. Postmenopausal women experience the highest risk of developing BC (3, 4), notwithstanding the menopause-associated estrogen withdrawal. The other endocrine hallmark of menopause is the stable elevation of gonadotrophins (5), but the role of these hormones in the incidence of post-menopausal diseases, including BC, has received little attention.

Follicle-stimulating hormone (FSH) and luteinizing hormone (LH) are glycoprotein hormones that control key steps of human and animal reproduction. LH and FSH exert these actions through receptors in the ovaries and testes, where they control steroidogenesis and gametogenesis (6). During the past decade, a scientific debate has arisen around the potential role of gonadotrophins on the development of BC (7–10). A number of studies have indicated that follicular-stimulating hormone receptor (FSHR) and luteinizing hormone receptor (LHR) are expressed in several cancers, suggesting that gonadotrophins may thus influence the development or progression of these diseases (11–15).

Our group has recently shown that LHR and FSHR are expressed and functional in several BC cell lines, in which they modulate migration and invasion through the control of rapidly acting signaling cascades triggered by G proteins on the cell membrane (15). Cancer cell motility and migration are critical steps needed to accomplish invasion and metastasis (16). Regulation of the actin cytoskeleton is key in this process; it is a tightly regulated event (17). In the presence of LH or FSH, the actin-binding protein moesin and the focal adhesion complex regulator focal adhesion kinase (FAK) are phosphorylated and then localize to the plasma membrane, where they endow cells with higher motility through increased adhesion to the extracellular matrix (15, 18). This is mostly driven by the recruitment of large intracellular protein complexes, such as the paxillin/cortactin/N-WASP-Arp2/3 complex, which promote the formation of molecular bridges between actin, integrins, and focal adhesion complexes at specialized cell membrane sites involved in cellular motility (17).

The aim of this study was to characterize the modulatory effects of FSH and LH on the expression of proteins related to BC cell motility and invasion and to couple these findings to functional assays. We explored the effects of different types of exposure to FSH and LH, such as consistently low, pre-pubertal-like amounts or consistently high, post-menopausal-like concentrations, and compared them to intermittent low/high amounts, representative of the variations in hormone levels that occur during reproductive years. We also studied the impact of LH on the expression of a broad set of genes related to cancer progression using a miniarray. Finally, we assessed the influence of LH on BC progression *in vivo*.

## Materials and Methods

### Cell Cultures and Treatments

The human breast carcinoma cell lines T-47D and MDA-MB-468 were obtained from the American Type Culture Collection. T-47D BC cells were grown in RPMI 1640 supplemented with l-glutamine (2 mM), 10% fetal bovine serum (FBS), penicillin and streptomycin under a 5% CO_2_ atmosphere at 37°C. MDA-MB-468 BC cells were incubated in L-15 medium Leibovitz containing 10% fetal calf serum and l-glutamine, penicillin, and streptomycin. Before experiments investigating genomic effects, the BC cell lines were kept 24 h in medium containing steroid-deprived FBS. LH (Luveris 75 IU) and FSH (GONAL-f 75 IU) were used with three different concentrations. LH and/or FSH were obtained from Merck Serono Laboratory. The concentrations were chosen to mimic pre-puberty (5 mUI/ml), an ovulatory pattern (5 + 50 mUI–40 h with 5 mUI/ml and 8 h with 50 mUI/ml) or postmenopause (50 mUI/ml).

### Animals

The study used 36 fertile healthy female Wistar rats (weight: 150–200 g), purchased from Harlan Nossan, Italy. They were divided into six groups of six rats each and were housed together in a climate-controlled room, with free access to food and water and 14 h of illumination (light on at 6:00 a.m. and light off at 8:00 p.m.). All experimental procedures were approved by the Ethical committee of the University of Pisa in accordance with the Guide for Care and Use of Laboratory Animals. Virgin female rats between 55 and 60 days old received a single subcutaneous injection of 14 mg N-nitroso-N-methylurea (NMU)/kg body weight (Sigma-Aldrich, St. Louis, MO, USA). NMU was dissolved in warm 0.9% NaCl acidified to pH 5.0 with acetic acid (vehicle). Control rats received a single i.p. injection of 0.9% NaCl solution as vehicle. After 15 weeks of NMU treatment, when 24 rats were ovariectomized (OVX) under a tiletamine plus zolazepam anesthesia (Zoletil, 1 mg/rat intramuscular). Ovariectomy was performed at the same estrous cycle stage, as indicated by daily vaginal smears. Active treatments were started 4 weeks after surgery. All animals were checked every other day for number of breast tumors and size of each tumor. FSH, LH, and estradiol were measured at baseline and after 30 and 60 days.

Groups were as follows:

(1) Fertile controls: 6 animals that had not been injected with NMU were used to control for potential NMU-related changes in FSH, LH, or estradiol levels over the 8-week-observation.
(2) Fertile NMU: 6 NMU-injected, sham-operated animals were used to check for hormonal changes and BC progression without any further intervention over a 8-week period.
(3) OVX NMU: 6 NMU-injected, ovariectomized animals were observed without any further intervention for 8 weeks for tumor progression and hormonal changes.
(4) OVX NMU + E2: 6 NMU-injected, ovariectomized animals received estradiol replacement (0.05 mg/kg/day) for 8 weeks.
(5) OVX NMU + LHRH analog: 6 NMU-injected, ovariectomized animals received a single injection of the LH analog Leuprorelin (0.05 IU/kg, which is maintained during a period of 30 days).
(6) OVX NMU + LHRH analog + E2: 6 NMU-injected, ovariectomized animals received a single injection of the LH analog Leuprorelin along with replacement with estradiol (0.05 mg/kg/day) for 8 weeks.

To evaluate the incidence and latency of tumor formation, we have palpated the thoracic and abdominal-inguinal mammary glands weekly starting at 4 weeks post-NMU injection and continued every other day for the whole 8-week intervention period with documentation of tumor number and size. Upon sacrifice at the end of the observation, a histological analysis of mammary gland and tumors was performed. Tumor incidence was determined as the number of animals with at least one palpable tumor or microscopic tumor lesion compared to the total number of animals in the group. Latency was calculated as the time between the NMU injection and the detection of the first palpable tumor.

### Immunoblottings

Cell lysates were separated by SDS-PAGE assay. Antibodies used were: FAK (dilution 1:1,000), p-FAK (Y397, 1:1,000), and moesin (clone 38, 1:1,000) (BD Transduction Laboratories, Lexington, KY, USA); Thr558-p-Moesin (sc-12895, 1:750), p-FAK-Tyr397 (sc-11765, 1:500), LHR (H-50, 1:500), FSHR (N-20, 1:500), and ACTIN (C-11, 1:1,000) (Santa Cruz Biotechnology, Santa Cruz, CA, USA). The blocking step was performed with 1% bovine serum albumin (BSA) solution for 30 min at room temperature. Primary Abs were incubated overnight and secondary Abs were incubated for 1 h with standard technique. Immunodetection was accomplished with enhanced chemiluminescence. Chemiluminescence was acquired with a quantitative digital imaging system (ChemiDoc™ XRS + System with Image Lab™ software #170-8265, Biorad, USA). The blots were quantified using the Image J software.

### Cell Immunofluorescence

T-47D BC cells were grown on coverslips. Cells were fixed for 30 min with paraformaldehyde (4%) and permeabilized for 5 min with 0.1% Triton. Blocking was performed with PBS containing 3% BSA for 30 min. BC cells were incubated with antibodies vs. phospho-FAK^Y397^ and phospho-Moesin^T558^ (1:500, BD Transduction Laboratories) overnight at 4°C followed by incubation with a fluorescein-conjugated goat anti-rabbit/mouse secondary antibody (1:200; Vector Laboratories). Texas Red-phalloidin (Sigma-Aldrich, St. Louis) was added for 30 min to reveal actin and the nuclei were counterstained with or 4′-6-diamidino-2-phenylindole (Sigma-Aldrich, St. Louis) and mounted with Vectashield mounting medium (Vector Laboratories, Burlingame, CA, USA). Immunofluorescence images were captured using a Nikon Eclipse E200 microscope (Japan) coupled to a high-resolution 590CU 5.0M CCD digital camera.

### Gene Silencing With RNA Interference

A synthetic small interfering RNA targeting FAK (siRNA SMARTpool FAK, MN-005607) and control siRNA (scrambled siRNA D-001810-01-05) was purchased from Dharmacon (Thermo Fisher Scientific Inc., USA); and two SureSilencing shRNA Plasmid Human LHR and FSHR and the negative control with the GFP marker (Cat KH01310G and KH07073G) were obtained from SuperArray Bioscience Corporation. As control, parallel cells were transfected with empty vector + plasmid with GFP (for shRNAs) and scrambled siRNAs (for siRNAs). The siRNA and plasmids were used at the final concentration of 50–75 nM to silence FAK, LHR, and FSHR according to the manufacturer’s instructions. T-47D cells (70–80% confluent) were transfected using Lipofectamine (Invitrogen) in Opti-MEM medium (Invitrogen) and treated 48 h after transfection. The efficacy of gene silencing was checked with western analysis and found to be optimal at 48 h.

### Moesin Silencing With Antisense Oligonucleotides

Moesin silencing was performed with a specific antisense phosphorothioate oligonucleotides (S-modified) (PONs, sequence 5′-TACGGGTTTTGCTAG-3′) complementary to the 1–15 position of the human moesin gene-coding region. As control, the complementary sense PON was used (5′-CTAGCAAAACCCGTA-3′). PONs transfections were performed in serum-free medium using Lipofectamine (Invitrogen, CA, USA) and added to the culture medium for 48 h at the final concentration of 50–75 nM. Moesin silencing was confirmed through western blot analysis 48 h after transfection.

### Cell Migration Assay

T-47D cells were plated in 6-well plate and grown to 70–80% confluence. Wounds were made in the monolayers by scratching the surface with a razor blade, pressing through the confluent T-47D cell monolayer into the plastic plate to mark the starting line. BC cells were swept away on one side of that line. Cells were washed, and 2.0 mL of RPMI 1640 containing steroid-deprived FBS added. Cytosine β-D-arabinofuranoside hydrochloride (Sigma) (10 µM), a selective inhibitor of DNA synthesis that does not inhibit RNA synthesis was used and replaced after 24 h to prevent cell proliferation (so to discern the actions of the gonadotrophins on movement from those on cell proliferation). Furthermore, absence of cell proliferation and viability of the cells were checked in preliminary experiments with MTT [3-(4,5-dimethylthiazol-2-yl)-2,5-diphenyltetrazolium bromide] tests. Migration was monitored for 48 h. Cells were digitally imaged and migration distance was measured by using phase-contrast microscopy and the distance was quantified using Image J Software.

### Cell Invasion Assay

Cell invasion was determined using the BD BioCoatTM growth factor reduced (GFR) MatrigelTM Invasion Chamber (BD Bioscience, USA). After rehydrating the Matrigel inserts, the test substance was added to the wells and the chambers were incubated for 48 h. After this, non-invading cells were discarded from the upper surface of the membrane using cotton-tipped swabs. Then the cells on the lower surface of the membrane were stained with Diff-Quick. Invading cells were observed and photographed under the microscope at 100× magnification (Nikon Eclipse E200 coupled to a high-resolution 590CU 5.0M CCD digital camera). Cells were counted in the central field of triplicate membranes.

### Bromodeoxyuridine (BrdU) Cell Proliferation Assay

The T-47D cells were plated at 5 × 10^4^ cells/well in RPMI 1640 with 10% FBS, penicillin, and streptomycin in 96-well culture plate. After seeding, the medium was changed to RPMI supplemented with 2% FBS, penicillin, and streptomycin. Then, the BC cells were cultured for 48 h, in the presence or absence of the following treatments: LH (5, 5 + 50, and 50 mUI/ml), FSH (5, 5 + 50, and 50 mUI/ml). BrdU incorporation into DNA was subsequently detected by immunoassay using a BrdU incorporation assay kit (Chemicon International). In brief, the cells were incubated at a concentration of 10 µM of BrdU for 4 h. After removal of the culture medium, the cells were fixed and incubated with anti-BrdU–peroxidase conjugate for 90 min. The cells were then incubated with a tetramethyl-benzidine substrate until color development was sufficient for photometric detection. The reaction was quantified by measuring absorbance using a spectrophotometer microplate reader set at dual wavelength of 450/550 nm (MULTISKAN EX; Thermo Scientific, Lafayette, CO, USA).

### Cell Treatment and RNA Preparation

T-47D BC cells were cultured as previously described in Section “Cell cultures and treatments.” After incubation for 24 h, the supernatant was removed, and the fresh RPMI 1640 containing LH in different concentration (5, 5 + 50, and 50 mUI/ml) was added into the cell culture, and the cells were incubated for further 48 h. After removing supernatant, RNA was extracted with the use of Total Isolation Kit (GA-013, SuperArray Bioscience Company) according to the manufacturer’s protocol. RNA quality was evaluated by running a sample with an RNA loading dye on a 1% agarose gel and inspecting for distinct 18S, 28S, and tRNA bands (Sigma-Aldrich), indicating lack of degradation; quantity was determined by A260 measurement. Samples were frozen at −80°C until use in SuperArray.

### Microarray Analysis

5 µg of RNA of human BC cells T-47D was reverse-transcribed with Biotin-16-dUTP (Roche Life Science) using the SuperArray TrueLabeling-AMP 2.0 kit (SuperArray, Inc., Frederick, MD, USA) according to the manufacturer’s instruction. The resulting biotinylated cDNA probe mixture was allowed to hybridize overnight to the Human BC Biomarker Gene Array (Cat no: OHS-402, SuperArray, Inc.) at 60°C overnight according to the manufacturer’s protocol. After washing and blocking array membranes, alkaline phosphatase-conjugated streptavidin was allowed to bind and the CDP-Star substrate (SuperArray, Inc., Frederick, MD, USA) chemiluminescence, and results were visualized. The results were quantified using a Quality One Analyzer software (Quantity One; BioRad, Hercules, CA, USA). All signal intensities were blank (negative control) DNA background subtracted, and normalized to the housekeeping gene actin-beta (ACTB) (positive control). Scatter plots were made from normalized signals. Relative gene expression levels were calculated as the ratio of the mean of all ACTB signals of all experiments and the mean of ACTB of each membrane. The relative expression level of each gene was based on the ratio of ACTB in untreated cells (CON). Changes in gene expression were illustrated as a fold increase/decrease. The cut-off induction determining expression was increased more than five times or less than five times. Genes which suited both the criteria were considered to be upregulated or downregulated. Statistical analysis of the data was performed using one-way analysis of variance (ANOVA) followed by Tukey–Kramer Multiple-Comparisons Test.

### Hormone Assays

Serum concentrations of LH, FSH, and estradiol (E2) were determined by immunoenzymatic method (ELISA). The measures are expressed as pmol/l for LH and FSH and nmol/l for E2. For each hormone, all samples were analyzed in the same assay and performed on different times, which corresponded to before E2 and/or LH analog administration (T_0_), at 30 (T_30_) and 60 (T_60_) days after E2 and/or LH analog administration.

### Tissue Harvest

Rat mammary glands were collected at 27 weeks post-treatment with NMU. In group 1–6 (*n* = 6), right abdominal-inguinal mammary glands were surgically removed by using anesthesia. Finally, these animals were sacrificed and the contralateral glands were collected.

### Immunohistochemistry

Tumors larger than 0.5 cm in diameter were removed from the mammary gland and fixed in 10% phosphate buffered formalin and processed for paraffin embedding as described previously (19). Both the primary tumors as well as the metastatic lesions underwent immunohistochemical staining using an antibody raised against LH and FSH receptor (LHR 1:200 and FSHR 1:150).

### Statistical Analysis

All values were expressed as mean ± SD. Statistical analyses and graphics were done using InStat from GraphPad Prism program version 5.0 Software (San Diego, CA, USA). Statistical differences between mean values were determined by ANOVA, followed by Tukey–Kramer Multiple-Comparisons test. Values of *P < 0.05 indicate significant differences between the groups. All values are expressed as mean ± SD.

## Results

### LH and FSH Regulate Moesin and FAK Protein Expression in BC Cells

**Figure 1:**
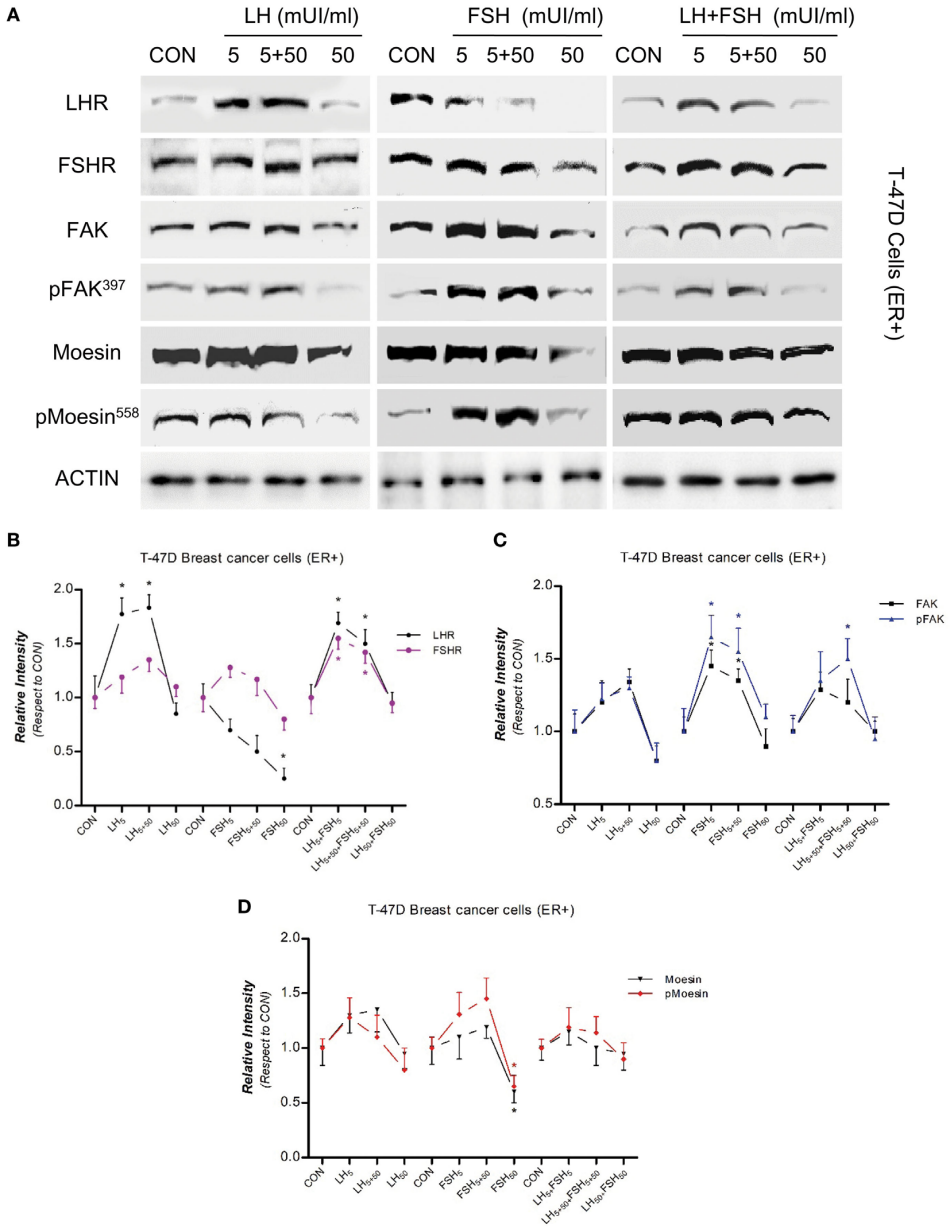

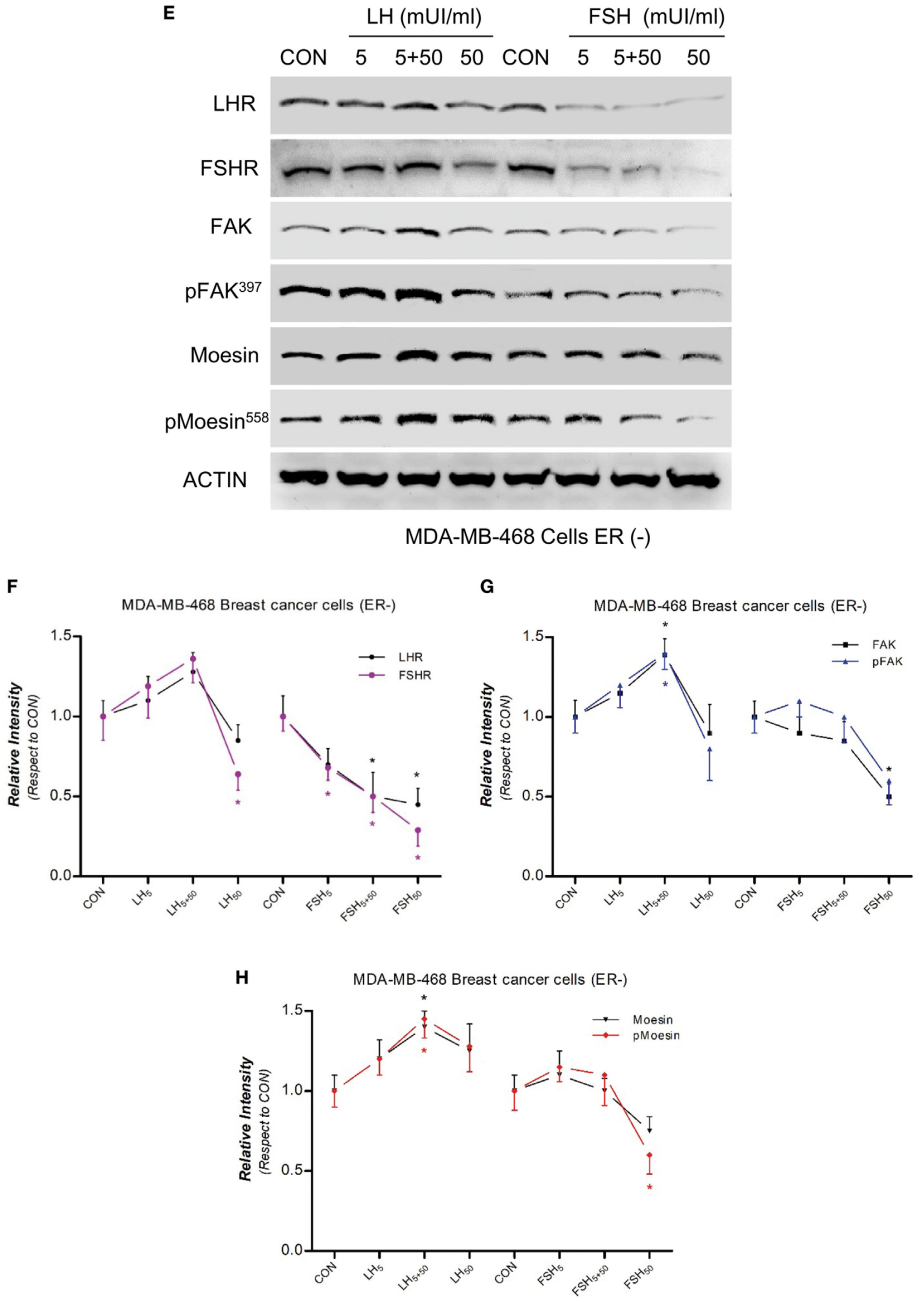
Modulatory effects of gonadotropins on breast cancer (BC) cell expression/activation of follicular-stimulating hormone receptor (FSHR), LHR, moesin, and focal adhesion kinase (FAK). Protein extracts from **(A)** T-47D (estrogen-receptor positive) and **(E)** MDAMB-468 (estrogen-receptor negative) BC cells treated for 48 h with increasing concentration of LH and follicle-stimulating hormone (FSH) (5, 5 + 50, and 50 mUI/ml) were assayed using western blot analysis for their overall content of LHR, FSHR, moesin, p-Moesin^T558^, FAK, p-FAKY^397^, and actin. **(B–D,F–H**) LHR, FSHR, moesin, p-Moesin^T558^, FAK, p-FAKY^397^ densitometry values were adjusted to actin intensity, respectively, then normalized to the control sample. *P < 0.05 vs. corresponding control. The experiments were performed in triplicates and representative images are shown.

We have recently demonstrated that multiple BC cell lines express functional LH and FSH receptors, including MCF-7, MDA-MB-231, and T-47D (15). Our first objective was, therefore, to determine the actions of LH and FSH exposure on the control of two actin cytoskeleton regulator proteins, moesin, and FAK, in BC cells. We treated T-47D (estrogen-receptor positive, ER+) BC cells for 48 h with three different concentrations of LH and/or FSH. The concentrations were chosen to mimic pre-puberty (5 mUI/ml), an ovulatory pattern (5 + 50 mUI/ml–40 h with 5 mUI/ml and 8 h with 50 mUI/ml), and postmenopause (50 mUI/ml), respectively. Moesin and FAK phosphorylation, which represents functional activation, and overall expression were increased by exposure to 5 or 5 + 50 mUI/ml with both gonadotrophins, whereas no increase or a frank decrease was seen with 50 mUI/ml of LH and/or FSH (Figures 1A–D). A parallel increase in LHR and FSHR expression was observed with 5 and 5 + 50 mUI/ml FSH or LH, with a decline to basal levels with 50 mUI/ml (Figures 1A–D). No additive effect was seen when gonadotrophins were administered simultaneously (Figures 1A–D).

We used estrogen-receptor negative (ER−) MDA-MB-468 BC cells to distinguish between the effects of gonadotrophin and estradiol. The response of LHR, FSHR, moesin, and FAK expression to LH exposure was more visible than that of FSH (Figures 1E–H).

We also examined the sub-cellular localization of p-moesin and p-FAK with immunofluorescence related to the exposure to LH and/or FSH (5 + 50 mUI/ml). BC cells treated with LH or FSH displayed p-moesin and p-FAK translocation to the membrane, accompanied by a membrane thickening (Figures S1A,B in Supplementary Material). Blockade of moesin or FAK with specific antisense PONs or siRNAs abrogated the gonadotrophin-induced membrane activation as well as p-moesin and p-FAK translocation to the plasma membrane.

### LH and FSH Promote BC Cell Migration

**Figure 2:**
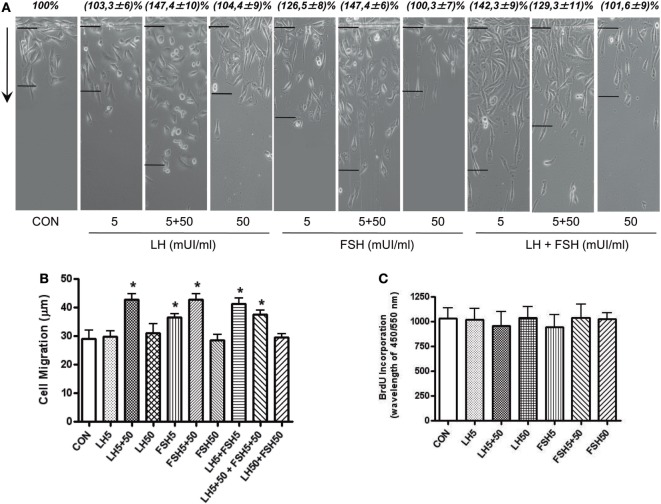
LH/follicle-stimulating hormone (FSH) promote breast cancer (BC) cellular migration. **(A)** T-47D BC cells were treated with LH and FSH in different concentrations (5, 5 + 50, and 50 mUI/ml) for 48 h. Horizontal cell migration was measured as the number of cells crossing the starting line or as the mean migration distance from the starting line. Sample images of horizontal migration in the different conditions are shown; the black lines indicate the mean migration distances. **(B)** Mean cell numbers or mean migration length is shown. Results are expressed as the mean ± SD of three separate experiments. *P ≤ 0.05 vs. control. **(C)** Effect of a 48-h exposure to LH and FSH on DNA synthesis of T-47D cells, as quantified by the bromodeoxyuridine assay. Data shown are representative of three independent experiments.

We performed horizontal migration assays to identify the relevance of moesin/FAK activation and expression induced by LH/FSH on BC cell movement. Treatment with LH and/or FSH (5 + 50 mUI/ml) for 48 h significantly increased the number of BC cells that migrated through the starting line, as well as the mean length of migration compared to control (Figures 2A,B).

In addition, we checked the action of LH and/or FSH on BC cell proliferation. Treatment with LH and FSH did not change cell proliferation at any of the concentrations tested (Figure 2C).

### LH and FSH Modulate Moesin-FAK Expression and Activation Through LHR and FSHR

**Figure 3:**
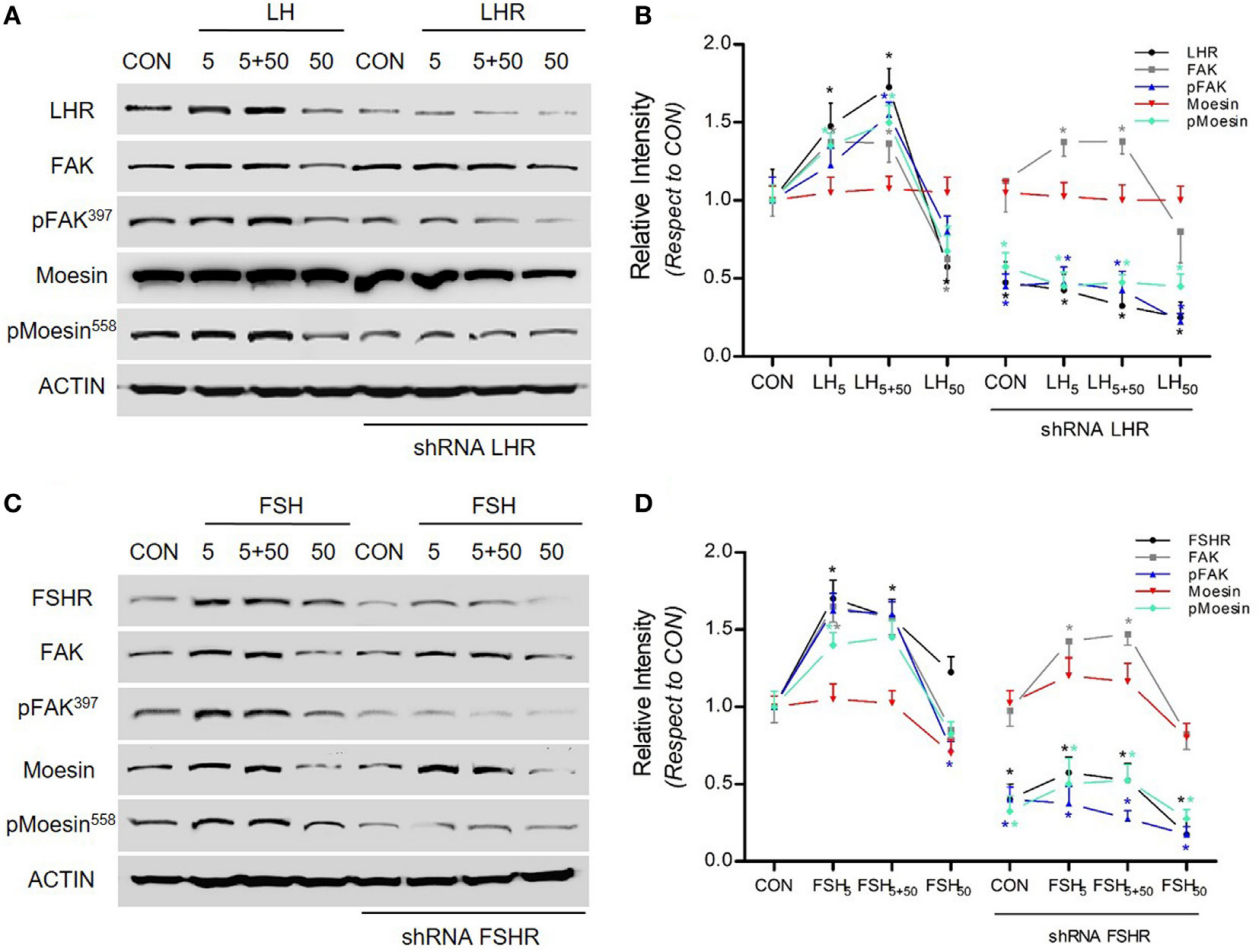
LH/follicle-stimulating hormone (FSH) signal to moesin and focal adhesion kinase (FAK) *via* LHR/follicle-stimulating hormone receptor (FSHR). **(A–C)** T-47D breast cancer cells were transfected with shRNA vs. LHR and FSHR (shRNA LHR and shRNA FSHR) or with vehicle, and protein analysis for LHR, FSHR, actin, wild-type (FAK and moesin), or p-FAK and p-moesin was performed on cell lysates after treatment with LH and FSH in different concentrations (5, 5 + 50, and 50 mUI/ml) for 48 h. **(B,D)** The graphs display the quantitative analysis of the intensity of the bands, obtained as number of photons measured by the ChemiDoc digital imaging system and evaluated with the Quantity One Software (Bio-Rad).

To determine whether the gonadotrophin receptors LHR and FSHR are involved in LH/FSH-induced moesin and FAK expression and activation, we blocked their expression with specific shRNA. Blockade of LHR and FSHR completely abolished LH/FSH-dependent moesin and FAK activation and expression in BC cells (Figures 3A–D).

### Intracellular Events Linking Regulation of LHR and FSHR to Cell Migration and Invasion

**Figure 4:**
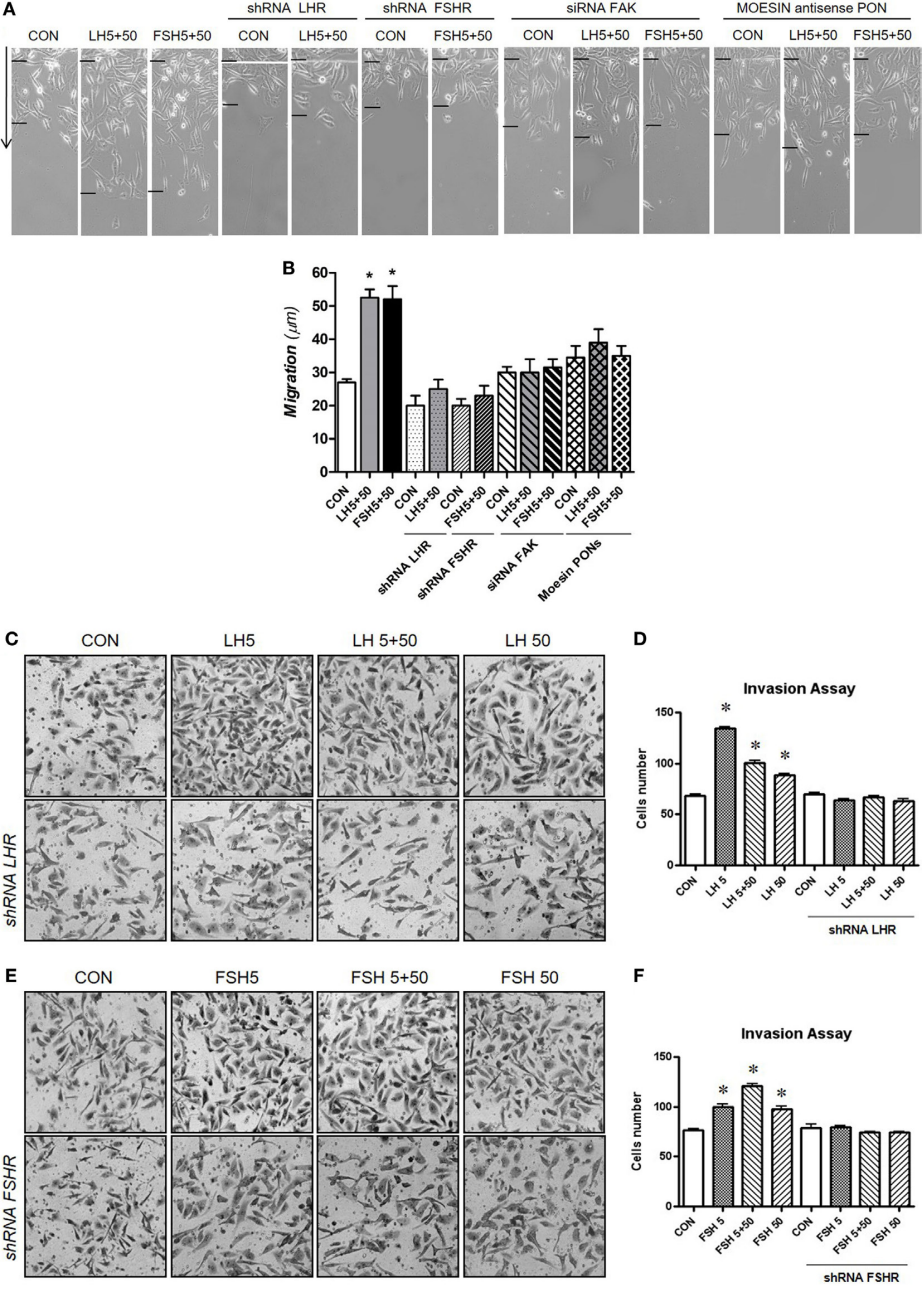

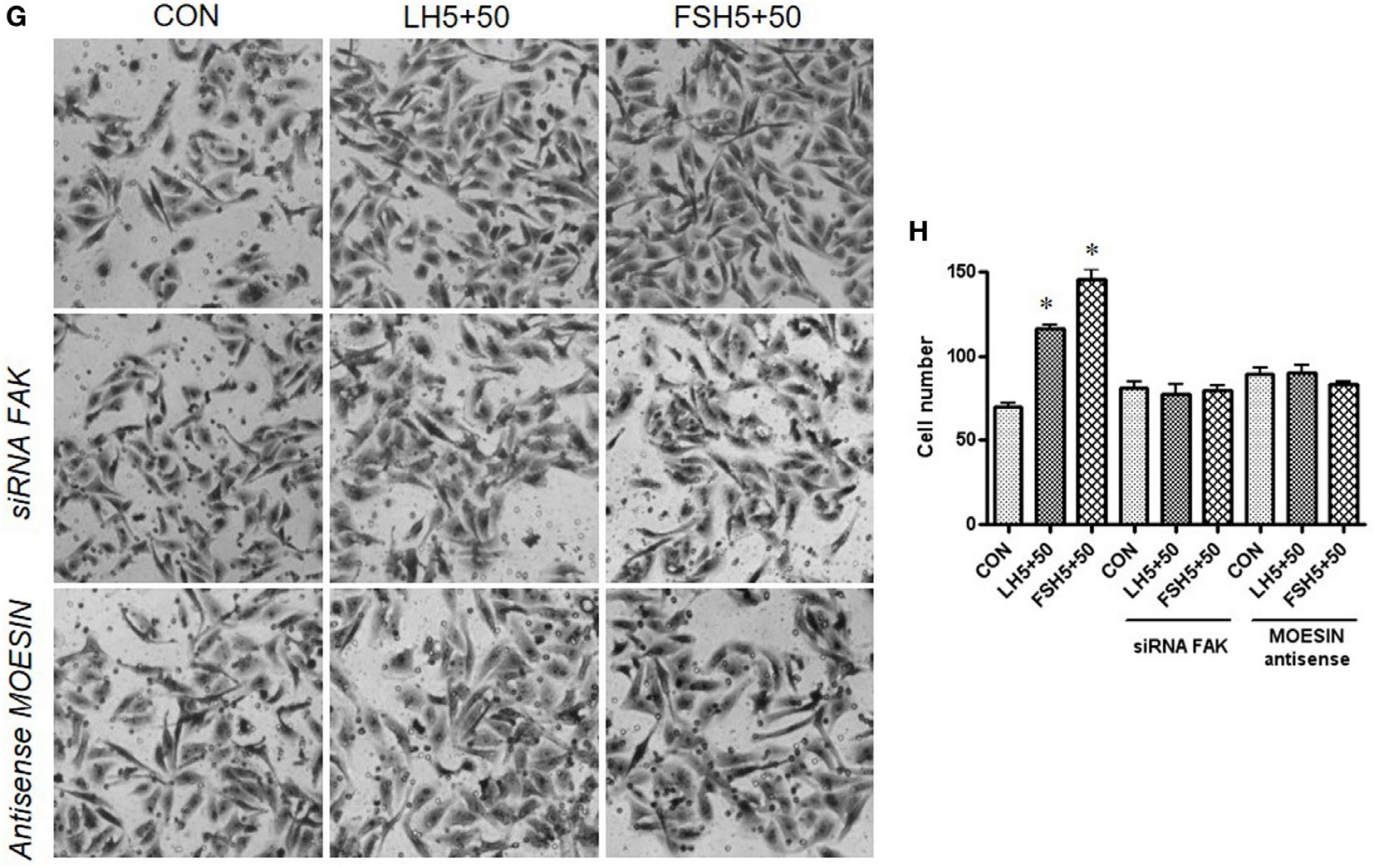
Gonadotrophins’ signaling to moesin and focal adhesion kinase (FAK) increase T-47D cell migration and invasion. Breast cancer cells were transfected with 100 nM target shRNA for LHR and follicular-stimulating hormone receptor (FSHR), siRNA for FAK and 75 nM target phosphorothioate oligonucleotides antisense vs. moesin for 48 h and then treated with LH and/or follicle-stimulating hormone (FSH) (5 + 50 mUI/ml) for 48 h. **(A,B)** Cell migration distances were measured as mean migration distance (μm) ± SD. *P ≤ 0.05 vs. control. The experiments were performed in triplicates and representative images are shown. **(C–H)** Cell invasion was assayed using invasion chambers. Average of invading cells observed, photographed under the microscope at 100× magnification and counted in three different central fields of triplicate membranes. *P ≤ 0.05 vs. control. The experiments were performed in triplicates. Invasion indexes and representative images are shown.

We further investigated the specific action of LHR/FSHR and moesin/FAK on BC cell migration and invasion. LH and FSH (5 + 50 mUI/ml) markedly increased horizontal migration (Figures 4A,B). Silencing of LHR and FSHR with specific shRNAs completely prevented this effect (Figures 4A,B). Increased BC cell migration induced by LH and FSH was also reduced by blocking moesin and FAK with specific antisense PON and siRNAs, respectively (Figures 4A,B). In addition, LH and FSH (5 + 50 mUI/ml) promoted BC cell invasion of a three-dimensional matrix (Figures 4C–H). The invasion induced by LH and/or FSH was prevented by silencing LHR, FSHR (Figures 4C–F), moesin, and FAK (Figures 4G,H).

### Effect of E2, LH, and FSH on Mammary Cancers in Female Rats

**Figure 5:**
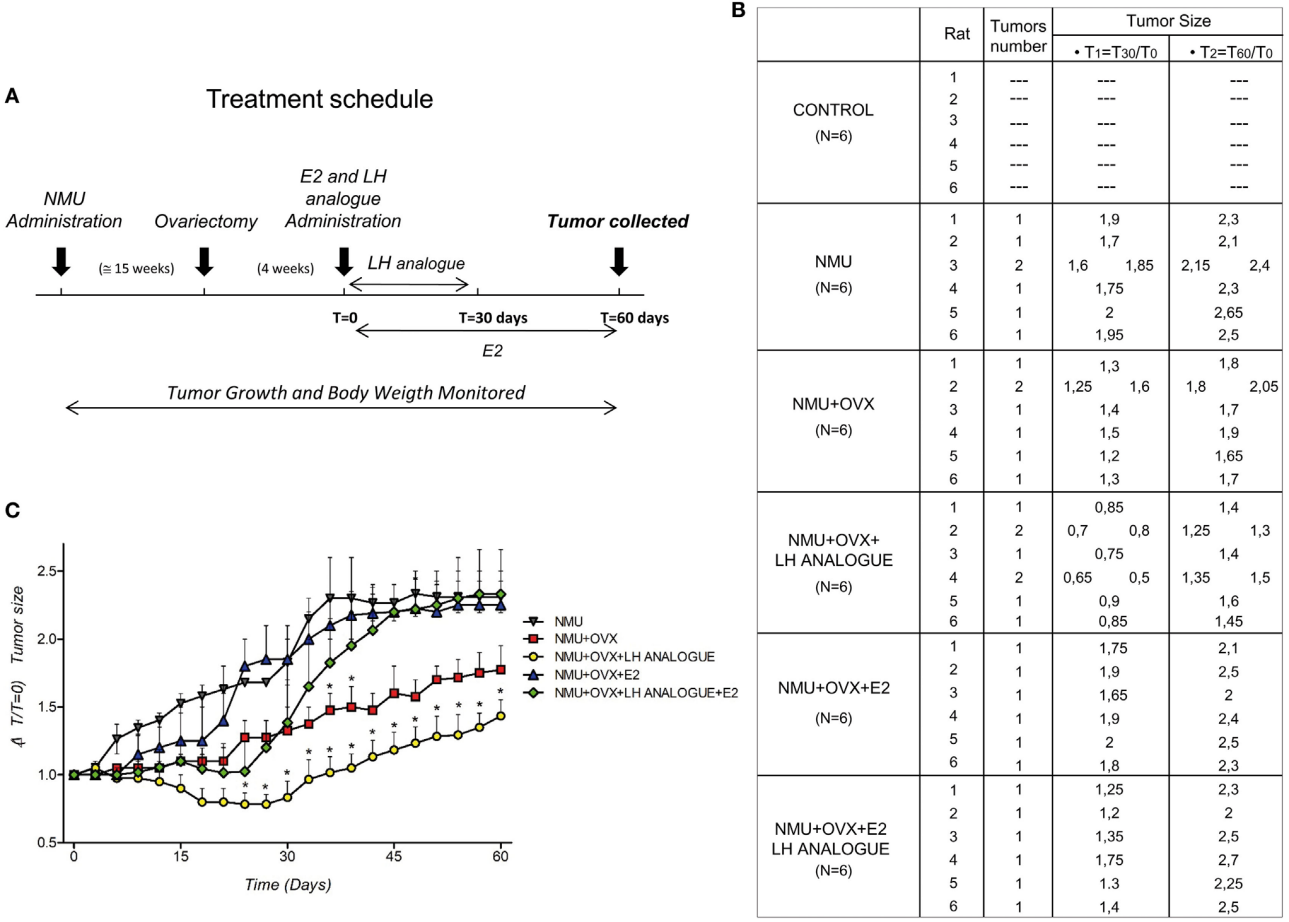

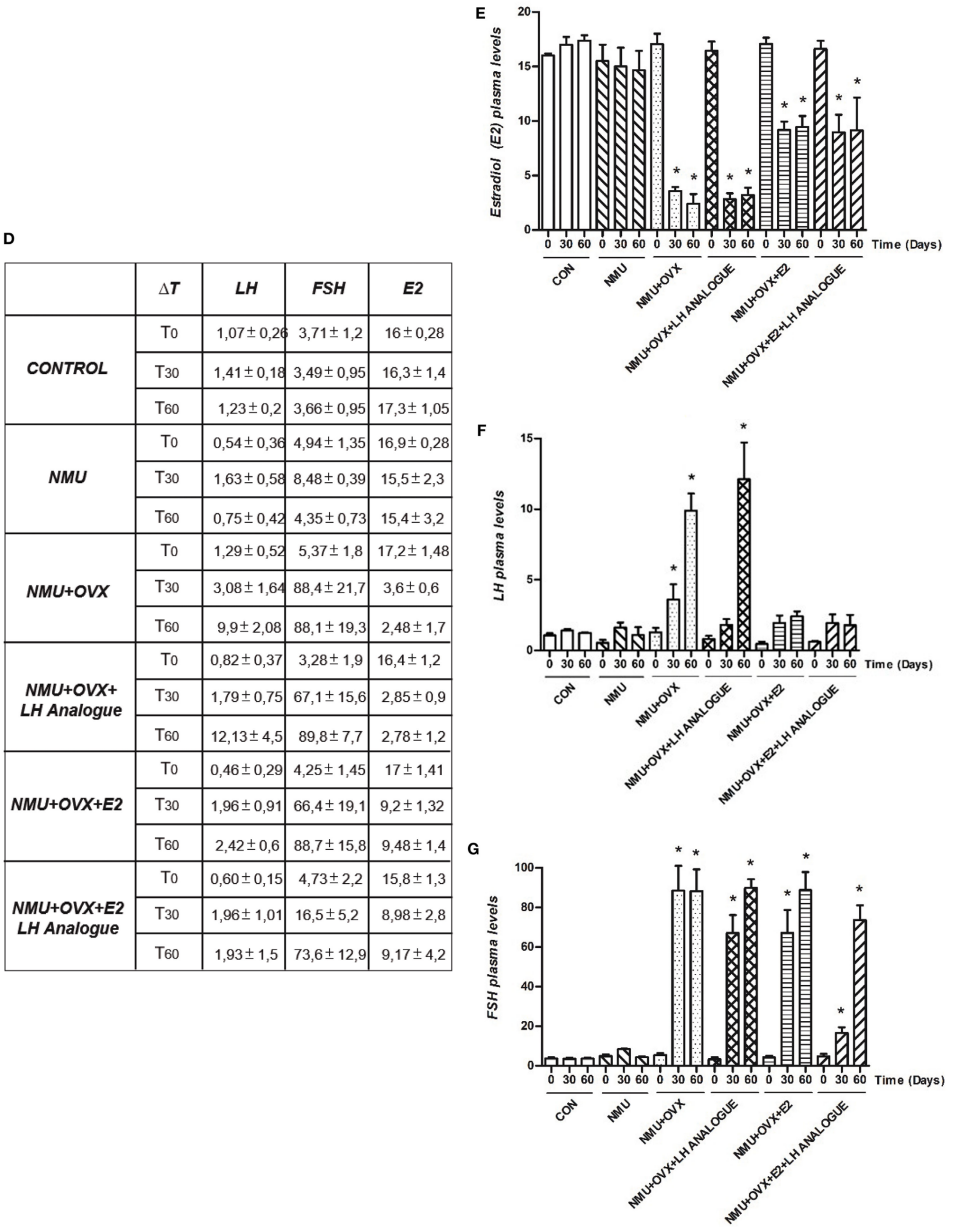
Regulation of E2 and LH in mammary tumors induced by N-nitroso-N-methylurea. **(A)** Reports a visual synthesis of the different treatment schedules in female rats. **(B)** Average tumors per rat, tumor number, and tumor size (ΔT1 = T_30_/T_0_ and ΔT2 = T_60_/T_0_) after 31 weeks of treatment in rats injected with NMU. **(C)** Tumor size vs. ΔT (ΔT = 60 days) was measured for each tumor. **(D–G)** Plasma levels (mean + SD) of E2 (nmol/l), LH (pmol/l), and follicle-stimulating hormone (FSH) (pmol/l) in fertile-control, NMU, NMU + OVX, NMU + OVX + LH analog, NMU + OVX + E2, or NMU + OVX + LH + E2 analog female rats. The statistical differences between mean values were determined by one-way analysis of variance (ANOVA), followed by Tukey–Kramer Multiple-Comparisons test. Values of *P < 0.05 indicate significant differences between NMU **(A–C)** and Control **(D–G)**.

In order to understand the *in vivo* action of LH and FSH in BC cells, we determined the regulation of LH, FSH, estradiol (E2), and Leuprorelin (LH analog) in mammary tumors induced by NMU, a chemical carcinogen in rats (Figure 5A). We used six experimental groups: (1) Rats treated with vehicle (Control); (2) Rats treated with NMU; (3) Rats treated with NMU and ovariectomized (OVX) to simulate a menopausal condition; (4) Rats treated with NMU, ovariectomized, and injected with Leuprorelin (to reduce LH levels); (5) Rats treated with NMU, ovariectomized, and treated with estradiol; and (6) Rats treated with NMU, ovariectomized, and injected with Leuprorelin and estradiol (Figure 5A). Twenty-seven weeks after the beginning of the protocol, we observed tumor formation in rats treated with NMU, NMU + OVX, NMU + OVX + LH analog, NMU + OVX + E2 and NMU + OVX + LH + E2 analog, but not in those injected with vehicle. We found a reduced tumor formation in the NMU + OVX group, but even more so with Leuprorelin (NMU + OVX + LH analog group). In the latter group, tumor size was significantly decreased compared to NMU alone (Figures 5B,C).

In parallel, we determined plasma levels of LH, FSH, and E2 in the study animals at different times (T_0_, T_30_, and T_60_, see Figure 5A) (Figure 5D). As expected, ovariectomized animals (NMU + OVX) had significantly lower E2 levels and higher LH and FSH concentrations (Figures 5D–G). E2 administration (NMU + OVX + E2) resulted in a normalization of E2 and LH plasma levels, whereas FSH was still slightly increased compared to controls (Figures 5D–G). Leuprorelin administration to OVX animals (NMU + OVX + LH analog) was associated with a reduced peripheral concentration of both gonadotrophins (Figures 5D–G). Additionally, administration of E2 in NMU + OVX + LH analog animals normalized the circulating levels of E2 but did not restore FSH levels (Figures 5D–J).

### Expression of LHR and FSHR in Mammary Tumors in Female Rats

**Figure 6:**
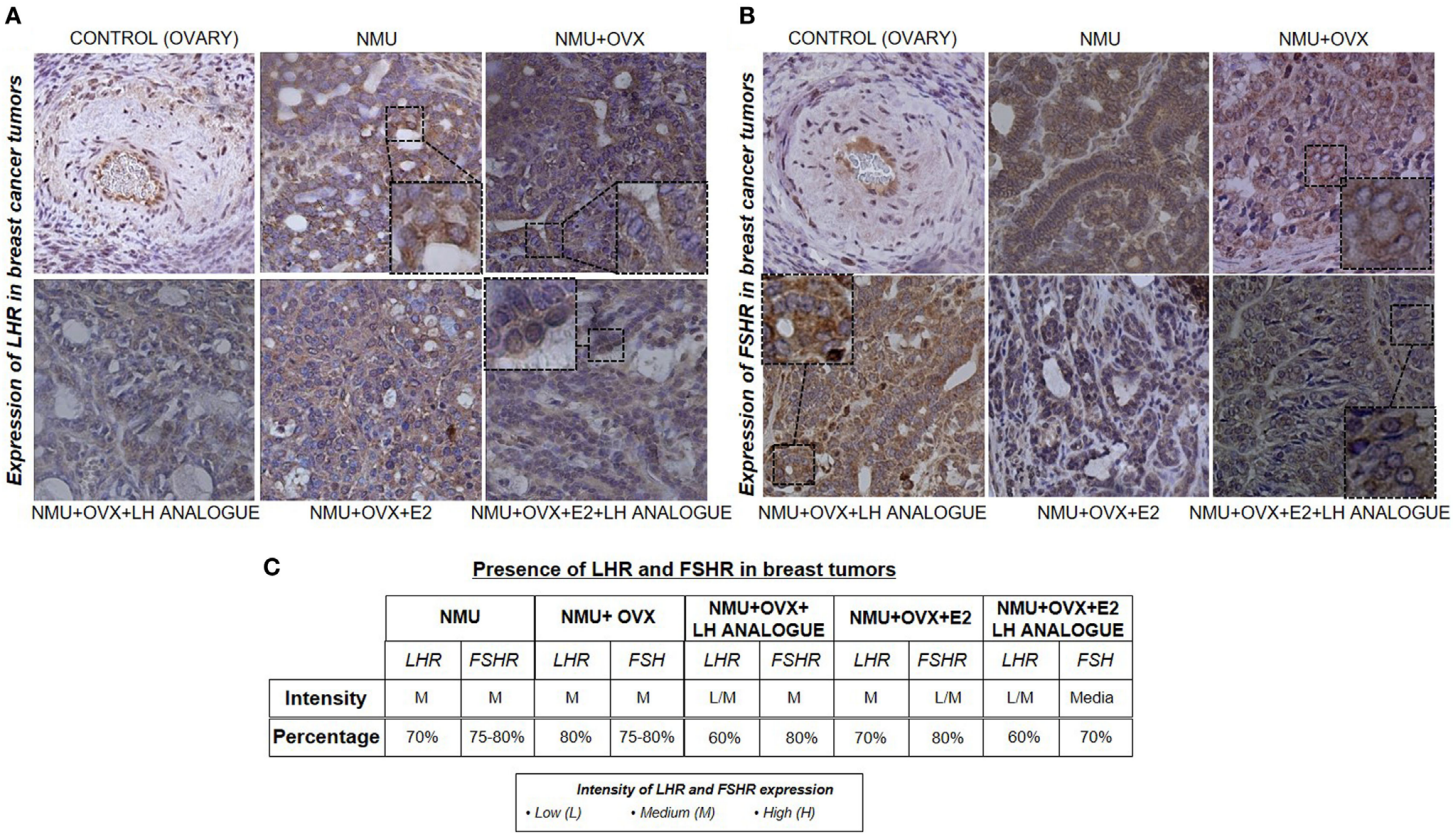
Expression of LHR and follicular-stimulating hormone receptor (FSHR) in breast tumors induced by N-nitroso-N-methylurea (NMU). **(A)** LHR and **(B)** FSHR expression in control (normal ovary tissue) and mammary tumors induced NMU in Wistar rats. Histological sections (4 mm) from normal ovary tissue and mammary tumors were used to identify the expression with immunochemistry. **(C)** Inmunohistochemical analysis of LHR and FSHR by means of the intensity of expression and percentage of cells with a positive stain in the different conditions in mammary tumors.

To understand the extragonadal regulation of LHR and FSHR in rat mammary tumors induced by NMU, we analyzed the expression of LHR and FSHR in rat ovary (internal control) and rat mammary tumors with immunohistochemistry. We found cytoplasmic expression of both receptors in all tumor specimens, particularly in epithelial but also in some stromal cells (Figures 6A–C).

### Different Expression Set for Cancer-Related Genes in Human BC Cells Upon Different Types of LH Treatment

**Figure 7:**
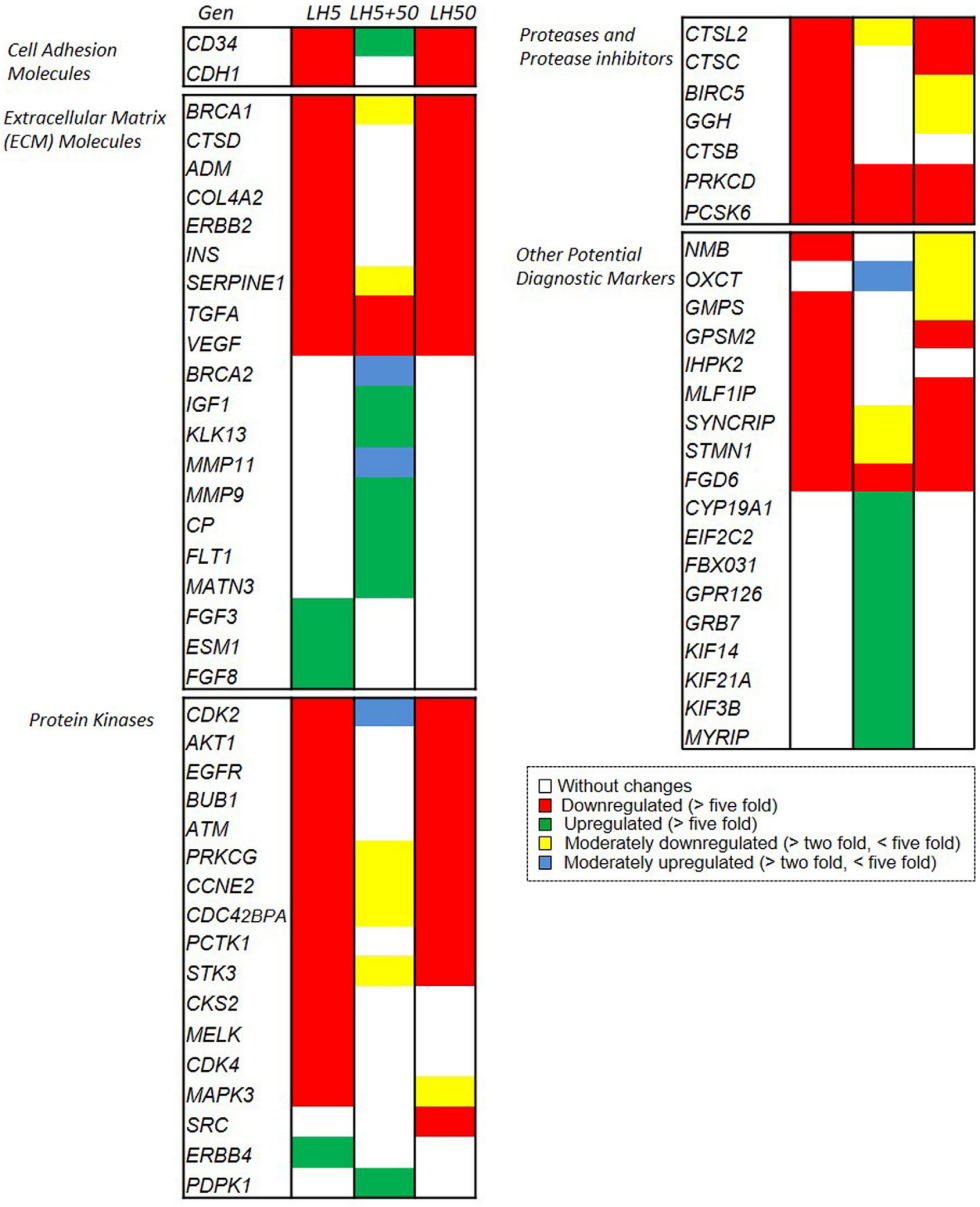
Microarray analysis of effects of LH on the expression of genes related to breast cancer biology. Different set of genes upregulated or downregulated, after LH treatment. Changes in gene expression are illustrated as fold increase/decrease. The cut-off induction determining expression was selected as an increase or decrease in expression of more than five times or more than two times as compared to untreated cells. Genes that suited the above criteria were considered to be upregulated or downregulated or to be moderately upregulated or downregulated, respectively, and they were arranged in different sets (Figures S1C–E in Supplementary Material).

Finally, we used a colorimetric miniarray targeting more than 100 genes of established relevance for cancer progression. Of all the genes studied (Supplementary Material), we further analyzed those closely related to BC progression (Figure 7).

The analysis of the results revealed a marked downregulation of genes related to migration–invasion after treatment with LH5 and LH50. These genes are involved in cell adhesion (CD34 and CDH1) or extracellular matrix regulation (such as ADM, INS, ERBB2, COL4A2, TGFa, etc.), protein kinases (CDK2, AKT1, ATM, EGFR, etc.), proteases etc., and are critical for cell motility (Figure 7, red colored genes). This downregulation suggests that the cells are losing the essential proteins to adhere to the substrate, providing a greater migratory and invasive phenotype.

The second noteworthy trend was an upregulation of other genes involved in extracellular matrix remodeling (MMP11, MMP9, MATN3) and of other protein kinases (KIF14, KIF21A, KIF3B, and MYRIP) after treatment with LH5 + 50 (Figure 7, green colored genes). The marked upregulation of the metalloproteinases MMP11 and MMP9 suggests that the extracellular matrix may have been actively degraded, thus favoring migration.

The results also revealed eight different sets of genes, grouped based on their expression levels, that were triggered by 48 h of treatment with LH according to the three previously described patterns (5, 5 + 50, and 50 mUI/ml, respectively) (Figures S1C–E in Supplementary Material).

## Discussion

Women <40 years usually have more aggressive BC tumors and a more unfavorable prognosis than older patients (20). Approximately one-fifth of affected women will die regardless of advances in screening, diagnosis, and treatment (21). Chemotherapy is fundamental in BC treatment although antineoplastic drugs may cause severe adverse and toxic effects (22). Although malignant breast tumors generally respond to initial chemotherapy, intrinsic or acquired multidrug resistance may restrict BC treatment and response to therapy (23, 24). Approximately 90% of deaths in BC patients occur through the development and growth of metastases at distant sites (25). It is, therefore, vital to develop alternative therapies to prevent or improve the prognosis of this disease.

The hormonal environment within the breast affects the development and progression of BC (26). We have recently demonstrated that increased LH and FSH levels could be essential to increase migration and invasion of BC cells (15). In the present study, we identified a novel signaling pathway by which LH and FSH receptors signal to moesin and FAK, which in turn could increase the ability of LHR/FSHR+ T-47D BC cells to migrate and invade the surrounding environment.

Cell motility is a complex and highly orchestrated process induced by multiple signaling molecules and implemented by actin rearrangement (27). The actin-binding protein moesin is critical for the reorganization of actin fibers that control the formation of membrane protrusions at the leading edge (15, 28–31). Cellular adhesion to the extracellular matrix also requires the development of focal adhesion complexes integrated by proteins, such as the integrin-binding proteins paxillin, vinculin, and talin, as well as c-Src, and FAK kinases (16). FAK phosphorylation/activation on Tyr397 is central for this process (32).

Our study revealed that LH and FSH regulate moesin and FAK expression and phosphorylation on Thr^558^ and Tyr^397^, respectively, leading to actin cytoskeleton remodeling and formation of focal adhesion complexes. FAK has recently been recognized as a main regulator of cell movement, particularly during tumor spread. This kinase is positively related to metastatic behavior. It is overexpressed in different types of cancer, such as endometrial (33), lung (34), melanoma (35), and ovarian (36). In human BC, FAK promotes cell motility and invasion *via* N-WASP (18), and increased levels of FAK expression are correlated with aggressive phenotype (37). In parallel, ablation of FAK activity in a rat BC metastasis model inhibited cancer dissemination to the lung (38). Blocking FAK in mouse and human mammary tumor cells induces cell senescence and loss of its invasive ability (39). These findings emphasize the importance of FAK activity and expression for cancer metastasis. The discovery of a control of FAK by gonadotrophins may thus offer novel insights to better understand the action of these hormones on BC metastasis.

It is also interesting to note that isolated BC cells respond in different ways to distinct patterns of exposure to FSH or LH. Both moesin and FAK activation, as well as the expression of a large set of genes related to BC growth and progression, are differentially modulated when FSH or LH are provided at continuously low (such as before puberty), continuously high (such as after menopause), or with an artificial sequence of a low concentration followed by a shorter exposure to a high concentration (grossly mimicking an ovulatory pattern). Indeed, gonadotrophins are known to act through pulsatile stimulation of their receptors in the ovary. This finding is, therefore, not surprising and consolidates the hypothesis that extragonadal gonadotrophin receptors may work similarly to those in the gonads.

In addition, we have shown that LHR and FSHR are also expressed in mammary tumors induced by NMU in rats. Their level of expression was constant in all experimental conditions (Figures 6A,C). One limitation of this experiment is the absence of LHR and FSHR immunostaining in mammary tissue of the control animals. It would be interesting to study whether the expression of both receptors is unregulated compared to a mammary cancerous tissue. Our *in vivo* results also indicate that the reduction of circulating amounts of gonadotrophins has a clear positive impact on breast tumor growth that is independent of estrogen concentrations.

In conclusion, this manuscript suggests that gonadotrophins play an important regulatory role on migration/invasion processes of BC cells and mammary tumors of female rats. This set of actions is related to the recruitment of cytoskeletal controllers and to a broader set of genes involved in BC progression. This observation is interesting in view of the fact that BC is more frequent in postmenopausal women in whom serum gonadotrophin levels are higher (40). If this were true, a space for using drugs that decrease the circulating amounts of FSH and LH, such as gonadotrophin-releasing hormone receptor antagonists (GnRH-R antagonists) may exist in postmenopausal women, with the potential to either decrease the development of BCs or to help in controlling cancer progression. Indeed, GnRH-R antagonists are commonly used in fertile women and in men to treat hormone-dependent tumors, but they are only thought to work through the secondary suppression of steroid synthesis (41–47). This has been confirmed by our animal work. Our study opens the possibility that FSH and LH suppression may be relevant *per se* in certain tumors. It would, therefore, be interesting to test the efficacy of these drugs in postmenopausal women.

## Ethics Statement

All experimental procedures were approved by the Ethical committee of the University of Pisa in accordance with the Guide for Care and Use of Laboratory Animals.

## Author Contributions

AMS carried out the majority of the experiments and drafted the manuscript. MF performed immunofluorescence and migration assays. SZ, ER, and AG performed *in vivo* experiments. PM performed western blot assays. AN performed immunohistochemistry experiments. AG was instrumental in funding the study and participated in the writing of the MS. TS planned and funded the project, supervised the experiments, and wrote the paper.

## Conflict of Interest Statement

The authors declare that the research was conducted in the absence of any commercial or financial relationships that could be construed as a potential conflict of interest.
