# Correction: Regulatory actions of LH and follicle-stimulating hormone on breast cancer cells and mammary tumors in rats

**DOI:** 10.3389/fendo.2025.1727490

**Published:** 2026-05-18

**Authors:** Angel Matias Sanchez, Marina Ines Flamini, Sara Zullino, Eleonora Russo, Andrea Giannini, Paolo Mannella, Antonio Giuseppe Naccarato, Andrea Riccardo Genazzani, Tommaso Simoncini

**Affiliations:** 1 Molecular and Cellular Gynecological Endocrinology Laboratory (MCGEL), Department of Clinical and Experimental Medicine, University of Pisa, Pisa, Italy; 2 Laboratorio de Transducción de Señales y Movimiento Celular, Instituto de Medicina y Biología Experimental de Cuyo (IMBECU), Consejo Nacional de Investigaciones Científicas y Técnicas (CONICET), Mendoza, Argentina; 3 Laboratorio de Biología Tumoral, Instituto de Medicina y Biología Experimental de Cuyo (IMBECU), Consejo Nacional de Investigaciones Científicas y Técnicas (CONICET), Mendoza, Argentina; 4 Department of Translational Research and of New Surgical and Medical Technologies, University of Pisa, Pisa, Italy

**Keywords:** LH, follicle stimulating hormone, moesin and focal adhesion kinase, cell motility, breast cancer

There was a mistake in **Figure 4A** as published. We observed a duplication of images. The original **Figure 4A** has been replaced with the original **Figure 4B**.

There was a mistake in **Figures 4C**–**F** as published. We observed a duplication of images and we have replaced the images with **Figures 4B-G**. The corrected **Figure 4** appears below.

**Figure 4:**
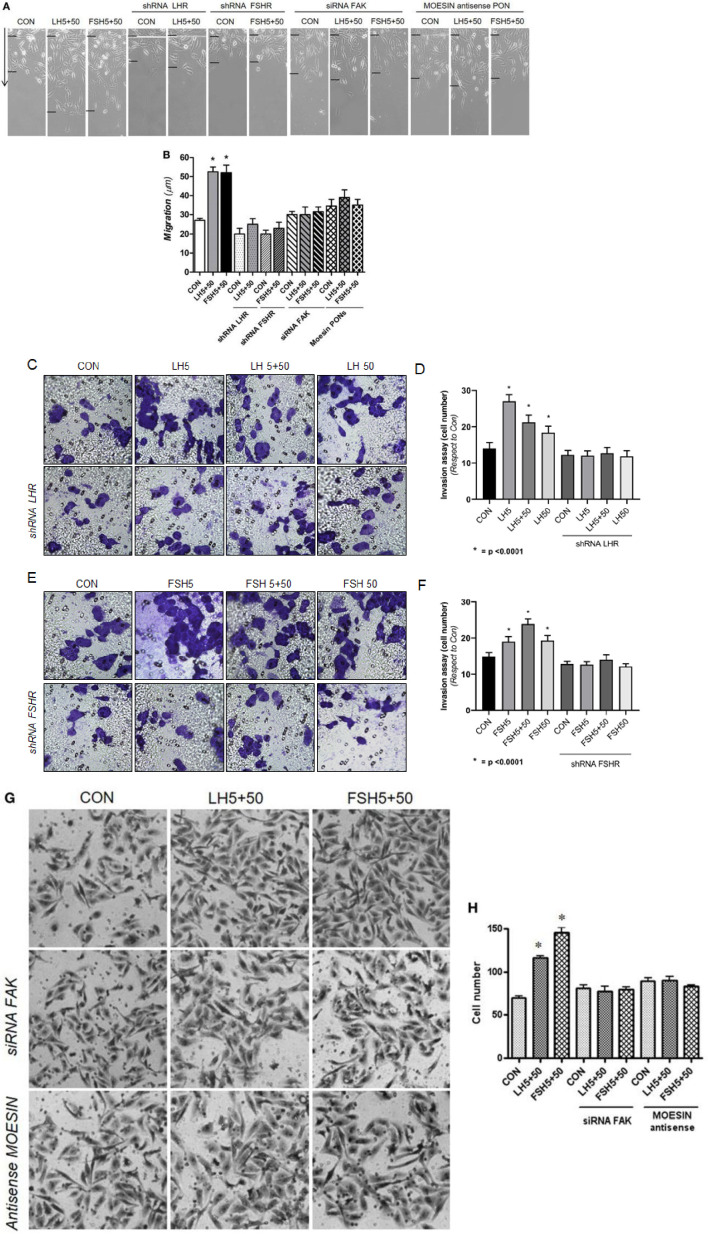
Gonadotrophins’ signaling to moesin and FAK increase T-47D cell migration and invasion. Breast cancer cells were transfected with 100 nM target shRNA for LHR and FSHR, siRNA for FAK and 75 nM target PONs antisense vs. moesin for 48 h and then treated with LH and/or FSH (5+50 mUI/ml) for 48 h. **(A)** Cell migration distances were measured as mean migration distance (µm) ± SD. *P ≤ 0.05 vs. Control. The experiments were performed in triplicates. **(B, D)** Cell invasion was assayed using Geltrex in invasion chambers. Average of invading cells observed, photographed under the microscope at 100× magnification and counted in three different central fields in triplicates. **(C, E)** The quantification of the invasion test and representative images are shown below. (*p< *0.0001*). **(F, G)** Cell invasion was assayed using Matrigel in invasion chambers. Average of invading cells observed, photographed under the microscope at 100× magnification and counted in three different central fields of triplicate membranes. *P ≤ 0.05 vs. control. The experiments were performed in triplicates. Invasion indexes and representative images are shown.

The original version of this article has been updated.

