# Supplementary material for: Regulatory Actions of LH and Follicle-Stimulating Hormone on Breast Cancer Cells and Mammary Tumors in Rats
Source: Front Endocrinol (Lausanne). 2018 May 16;9:239. doi: 10.3389/fendo.2018.00239 (PMC5964138; doi:10.3389/fendo.2018.00239)
Supplement: Supplementary file 1 [file presentation_1.PDF]

Suplemental data online

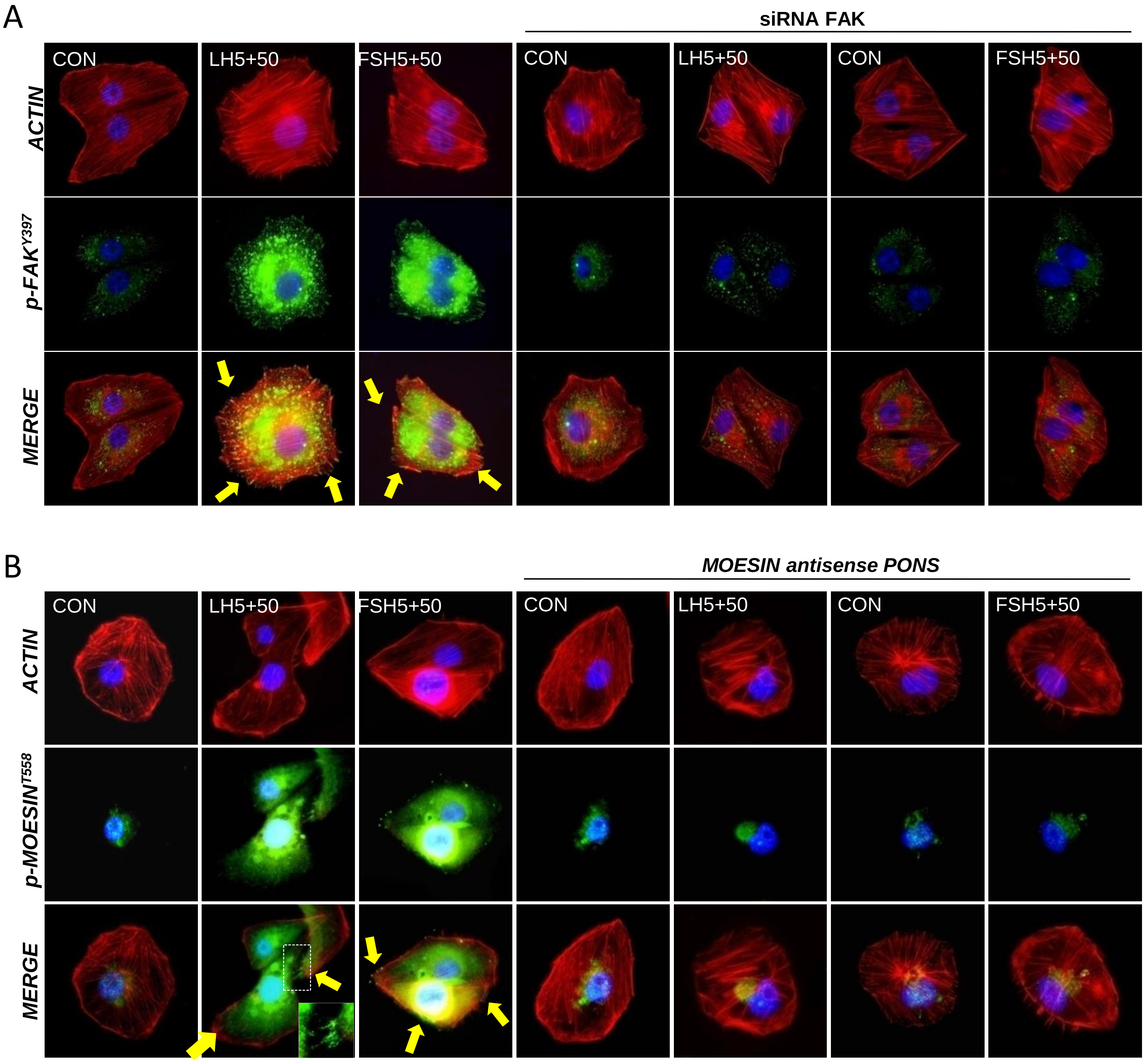


Phosphorylation and membrane translocation of p-FAK and p-Moesin. BC cells were exposed for 20 min to 5+50 mIU/mL of either LH and/or FSH, with or without the specific action of transfected siRNAs vs FAK and anti-sense moesin oligonucleotides. For details see the methods section.

C

| Profiles | CON | $LH_5$ | $LH_{5+50}$ | $LH_{50}$ |
|---|---|---|---|---|
| 1 | **** | | **** | |
| 2 | **** | * | ***** | ** |
| 3 | | | *** | |
| 4 | **** | * | ** | ** |
| 5 | *** | | | |
| 6 | | *** | | |
| 7 | * | * | * | |
| 8 | * | * | * | * |

Proportion of different set of genes, grouped with respect to their expression levels, triggered after LH treatment.

D

TABLE I – ***FUNCTIONAL CLASSIFICATION AND REPRESENTATIVE EXAMPLES OF GENES UPREGULATED AND DOWNREGULATED (more that five fold)***

| **Set 1** | **Functional classification** | |
|---|---|---|
| *Cell cycle* | *Cell Adhesion Molecules* | *Proteases and Protease Inhibitors* |
| BRCA1 | BRCA1 | CTSD |
| CCNE1 | *Apoptosis* | *Others Potential Markers* |
| CCNB2 | MX1 | DCK |
| CENPF | BAG1 | AP2B1 |
| MAD2L1 | MYBL2 | KRT19 |
| *Cell Grown and Proliferation* | BRCA1 | PALM2-AKAP2 |
| BMP6 | *Protein Kinases* | ACADS |
| CDK2 | CDK2 | AKAP1 |
| CSF1 | BUB1 | NMU |
| CHPT1 | MELK | GCN1L1 |
| EGFR | *Transcription Factor and Regulators* | GNAZ |
| BUB1 | FOS | |
| *Extracellular Matriz (ECM)* | JUN | |
| CTSD | HMGB3 | |
| ADM | MYBL2 | |

| **Set 2** | | |
|---|---|---|
| *Cell cycle* | *Apoptosis* | *Proteases and Protease Inhibitors* |
| MYC | TP53 | CTSC |
| BIRC5 | AKT1 | BIRC5 |
| BUB1 | BAG3 | GGH |
| CKS2 | BCL2L1 | *Others Potential Markers* |
| IGF2 | BIRC5 | NMB |
| CCNB1 | BNIP3 | OXCT |
| *Cell Grown and Proliferation* | RAD21 | GMPS |
| AR | *DNA Repair* | GPSM2 |
| MYC | TP53 | IHPK2 |
| CKS2 | RAD21 | MLF1IP |
| IGF2 | *Protein Phosphatases* | CTPS |
| *Cell Differentiation* | BIRC5 | DEGS |
| NDRG1 | GGH | EGLN1 |
| *Extracellular Matriz (ECM)* | *Transcription Factor and Regulators* | CA9 |
| IGF2 | AR | CD68 |
| *Protein Kinases* | MYC | CYC1 |
| AKT1 | TP53 | VIM |
| CKS2 | TSG101 | BBC3 |

| Set 3 | Functional classification |
|---|---|
| *Cell cycle*<br>BCL2 | |
| **Set 4** | |
| *Cell cycle*<br>TP53<br>TSG101<br>*Transcription Factors and Regulators*<br>TBX3 | Proteases and Protease Inhibitors<br>UCHL5<br>*Others Potential Markers*<br>RRAGD<br>TK1<br>ABCB1<br>NUSAP1 |
| **Set 5** | |
| *Cell Grown and Proliferation*<br>TGFA | *Others Potential Markers*<br>SYNCRIP<br>STMN1 |
| **Set 6** | |
| *Cell cycle*<br>FGF3<br>*Cell Grown and Proliferation*<br>ESM1<br>ERBB4 | *Angiogenesis Factors*<br>FGF3<br>*Protein Kinases*<br>ERBB4 |
| **Set 7** | |
| *Cell cycle*<br>BAX<br>*Protein Kinases*<br>SRC | *Apoptosis*<br>BAX<br>RFC4 |
| **Set 8** | |
| *Cell cycle*<br>RB1 | *Transcription Factors and Regulators*<br>RB1 |

Table I. Different expression set for cancer-related genes in human breast cancer cells upon different types of LH treatment according to the three previously described patterns (5, 5+50 and 50 mUI/ml). See the protocol in the Material and methods section.

E

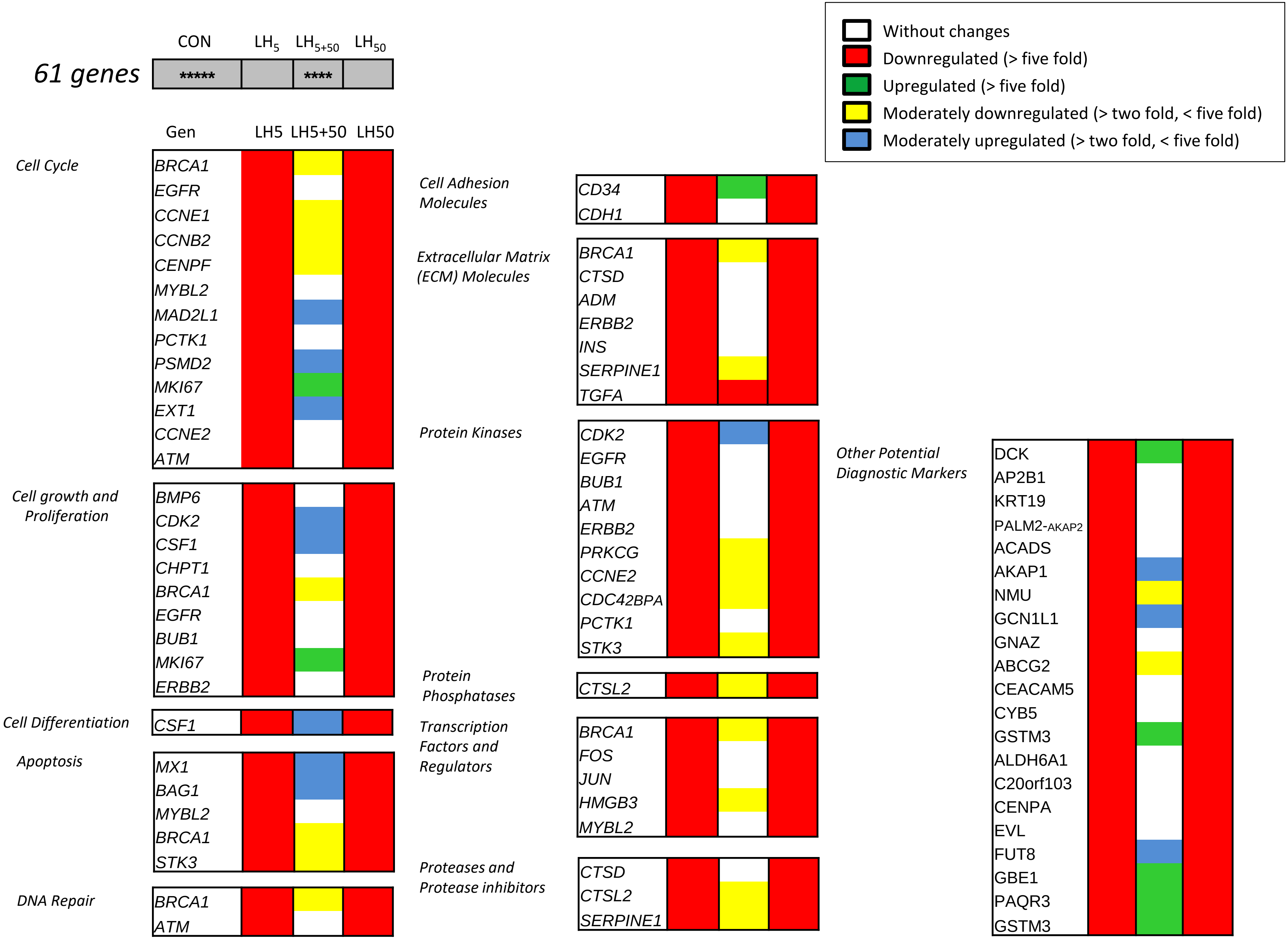

CON
LH5
LH5+50
LH50
61 genes
*****
****
Without changes
Downregulated (> five fold)
Upregulated (> five fold)
Moderately downregulated (> two fold, < five fold)
Moderately upregulated (> two fold, < five fold)
Gen
LH5
LH5+50
LH50
Cell Cycle
BRCA1
EGFR
CCNE1
CCNB2
CENPF
MYBL2
MAD2L1
PCTK1
PSMD2
MKI67
EXT1
CCNE2
ATM
Cell growth and Proliferation
BMP6
CDK2
CSF1
CHPT1
BRCA1
EGFR
BUB1
MKI67
ERBB2
Cell Differentiation
CSF1
Apoptosis
MX1
BAG1
MYBL2
BRCA1
STK3
DNA Repair
BRCA1
ATM
Cell Adhesion Molecules
CD34
CDH1
Extracellular Matrix (ECM) Molecules
BRCA1
CTSD
ADM
ERBB2
INS
SERPINE1
TGFA
Protein Kinases
CDK2
EGFR
BUB1
ATM
ERBB2
PRKCG
CCNE2
CDC42BPA
PCTK1
STK3
Protein Phosphatases
CTSL2
Transcription Factors and Regulators
BRCA1
FOS
JUN
HMGB3
MYBL2
Proteases and Protease inhibitors
CTSD
CTSL2
SERPINE1
Other Potential Diagnostic Markers
DCK
AP2B1
KRT19
PALM2-AKAP2
ACADS
AKAP1
NMU
GCN1L1
GNAZ
ABCG2
CEACAM5
CYB5
GSTM3
ALDH6A1
C20orf103
CENPA
EVL
FUT8
GBE1
PAQR3
GSTM3

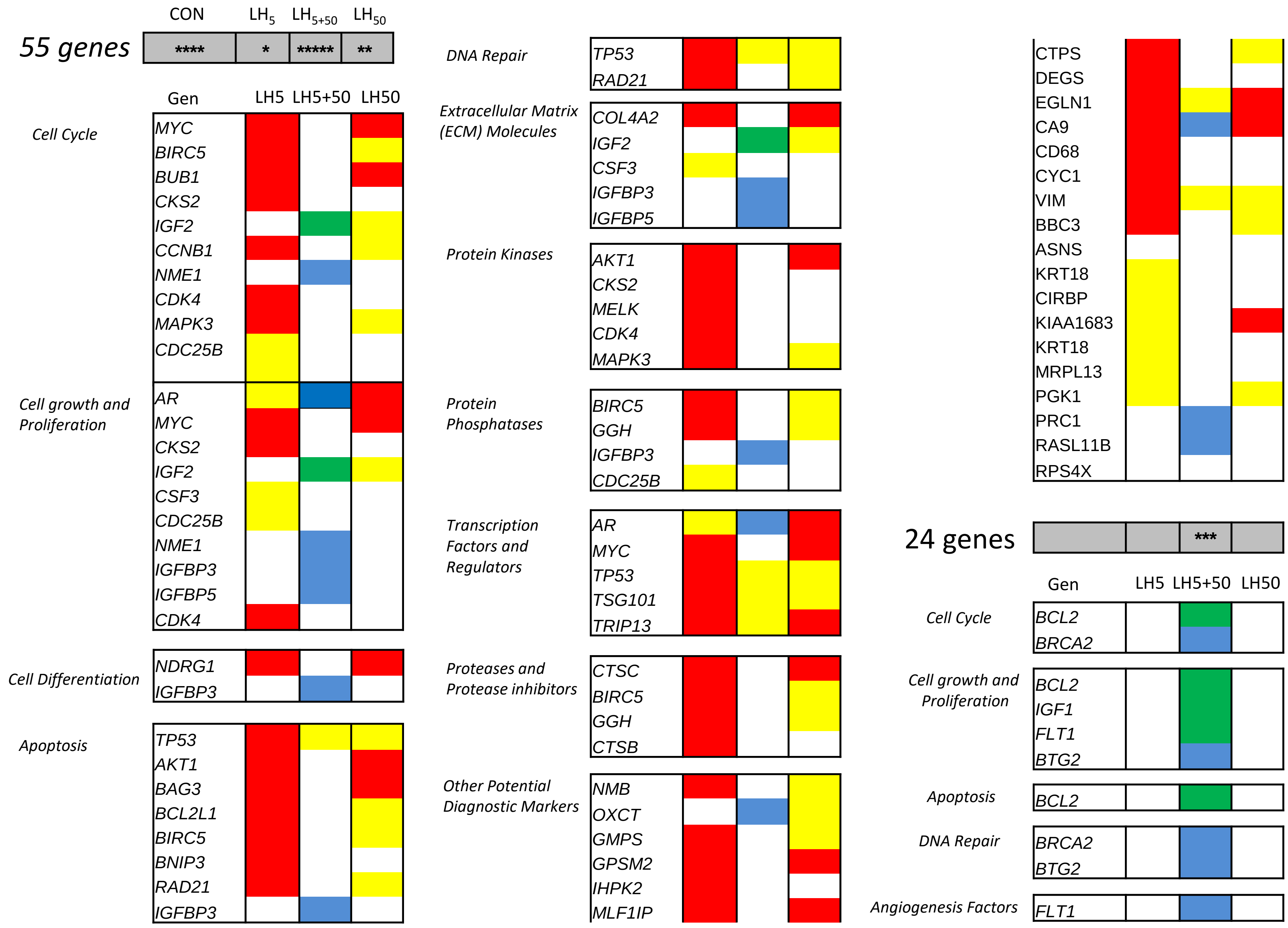
55 genes
CON
$LH_5$
$LH_{5+50}$
$LH_{50}$
****
*
*****
**
Gen
LH5
LH5+50
LH50
Cell Cycle
MYC
BIRC5
BUB1
CKS2
IGF2
CCNB1
NME1
CDK4
MAPK3
CDC25B
Cell growth and Proliferation
AR
MYC
CKS2
IGF2
CSF3
CDC25B
NME1
IGFBP3
IGFBP5
CDK4
Cell Differentiation
NDRG1
IGFBP3
Apoptosis
TP53
AKT1
BAG3
BCL2L1
BIRC5
BNIP3
RAD21
IGFBP3
DNA Repair
TP53
RAD21
Extracellular Matrix (ECM) Molecules
COL4A2
IGF2
CSF3
IGFBP3
IGFBP5
Protein Kinases
AKT1
CKS2
MELK
CDK4
MAPK3
Protein Phosphatases
BIRC5
GGH
IGFBP3
CDC25B
Transcription Factors and Regulators
AR
MYC
TP53
TSG101
TRIP13
Proteases and Protease inhibitors
CTSC
BIRC5
GGH
CTSB
Other Potential Diagnostic Markers
NMB
OXCT
GMPS
GPSM2
IHPK2
MLF1IP
CTPS
DEGS
EGLN1
CA9
CD68
CYC1
VIM
BBC3
ASNS
KRT18
CIRBP
KIAA1683
KRT18
MRPL13
PGK1
PRC1
RASL11B
RPS4X
24 genes
***
Gen
LH5
LH5+50
LH50
Cell Cycle
BCL2
BRCA2
Cell growth and Proliferation
BCL2
IGF1
FLT1
BTG2
Apoptosis
BCL2
DNA Repair
BRCA2
BTG2
Angiogenesis Factors
FLT1

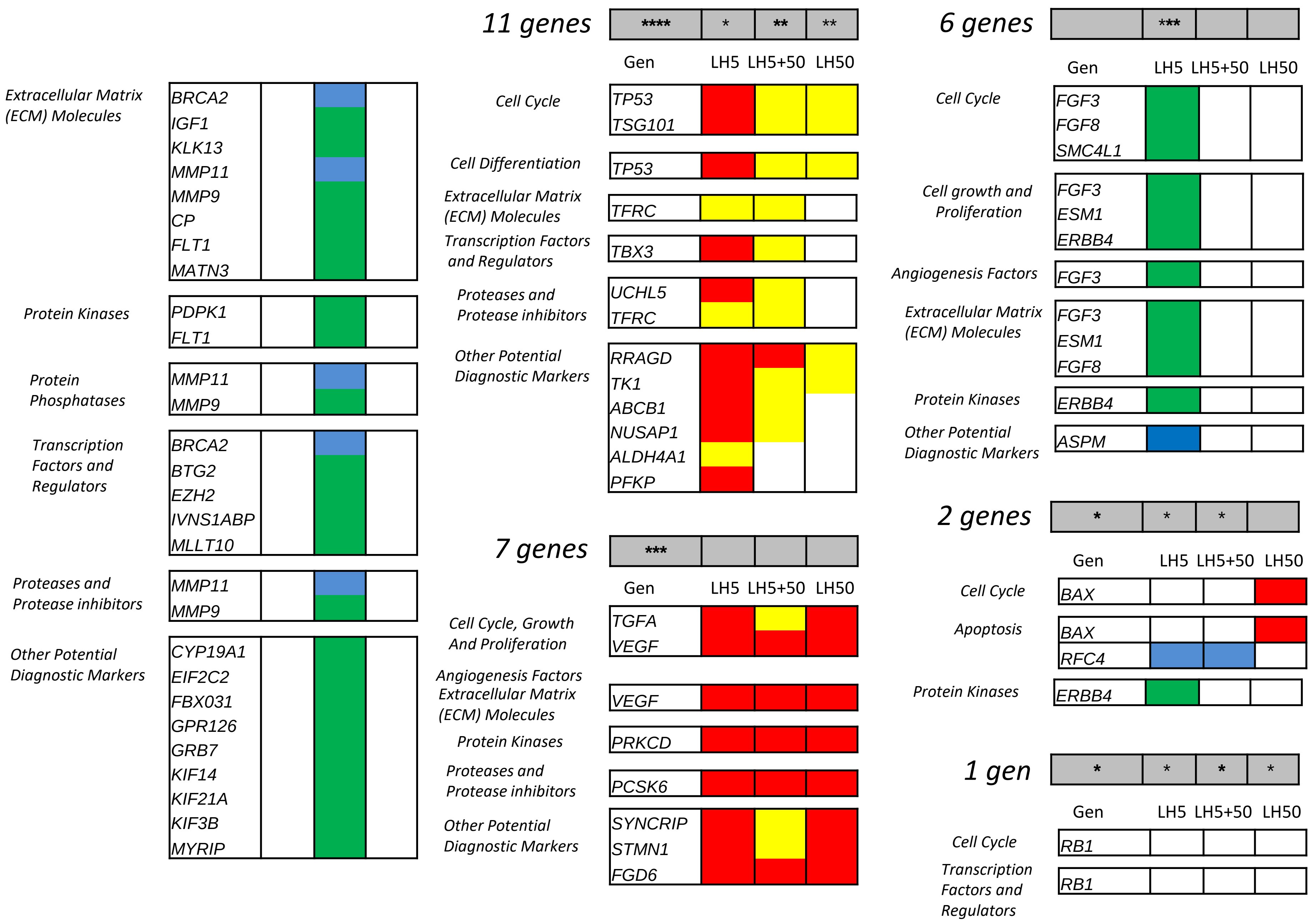

11 genes
****
*
**
**
Gen
LH5
LH5+50
LH50
Cell Cycle
TP53
TSG101
Cell Differentiation
TP53
Extracellular Matrix (ECM) Molecules
TFRC
Transcription Factors and Regulators
TBX3
Proteases and Protease inhibitors
UCHL5
TFRC
Other Potential Diagnostic Markers
RRAGD
TK1
ABCB1
NUSAP1
ALDH4A1
PFKP
7 genes
***
Gen
LH5
LH5+50
LH50
Cell Cycle, Growth And Proliferation
TGFA
VEGF
Angiogenesis Factors Extracellular Matrix (ECM) Molecules
VEGF
Protein Kinases
PRKCD
Proteases and Protease inhibitors
PCSK6
Other Potential Diagnostic Markers
SYNCRIP
STMN1
FGD6
Extracellular Matrix (ECM) Molecules
BRCA2
IGF1
KLK13
MMP11
MMP9
CP
FLT1
MATN3
Protein Kinases
PDPK1
FLT1
Protein Phosphatases
MMP11
MMP9
Transcription Factors and Regulators
BRCA2
BTG2
EZH2
IVNS1ABP
MLLT10
Proteases and Protease inhibitors
MMP11
MMP9
Other Potential Diagnostic Markers
CYP19A1
EIF2C2
FBX031
GPR126
GRB7
KIF14
KIF21A
KIF3B
MYRIP
6 genes
***
Gen
LH5
LH5+50
LH50
Cell Cycle
FGF3
FGF8
SMC4L1
Cell growth and Proliferation
FGF3
ESM1
ERBB4
Angiogenesis Factors
FGF3
Extracellular Matrix (ECM) Molecules
FGF3
ESM1
FGF8
Protein Kinases
ERBB4
Other Potential Diagnostic Markers
ASPM
2 genes
*
*
*
Gen
LH5
LH5+50
LH50
Cell Cycle
BAX
Apoptosis
BAX
RFC4
Protein Kinases
ERBB4
1 gen
*
*
*
*
Gen
LH5
LH5+50
LH50
Cell Cycle
RB1
Transcription Factors and Regulators
RB1
